# The inoculum effect of methicillin-susceptible *Staphylococcus aureus* on cefazolin and other antimicrobial agents

**DOI:** 10.1128/spectrum.00333-26

**Published:** 2026-07-23

**Authors:** Aikuang Ma, Linlin Xu, Lianyan Xie, Shuzhen Xiao, Feifei Gu, Qing Chen, Kang Xu, Jingyong Sun

**Affiliations:** 1 Department of Laboratory Medicine, Ruijin Hospital, Shanghai Jiao Tong, University School of Medicine 66281, Shanghai, China; 2 Department of Laboratory Medicine, Electric Power Hospital 726362 , Shanghai, China; Houston Methodist Hospital, Houston, Texas, USA

**Keywords:** methicillin-susceptible *Staphylococcus aureus*, inoculum effect, cefazolin, antibacterial agents

## Abstract

The inoculum effect (IE) refers to a reduced susceptibility of methicillin-susceptible *Staphylococcus aureus* (MSSA) to certain antibiotics under high bacterial inocula and may contribute to treatment failure. This exploratory study assessed IE prevalence among 234 nonduplicate MSSA isolates across 11 agents spanning major therapeutic classes, including cefazolin, and characterized IE-positive clones via whole-genome sequencing to inform clinical strategies. Minimum inhibitory concentrations (MICs) were determined by broth microdilution at standard and high inocula. Whole-genome sequencing was performed on IE-positive strains to identify β-lactamase types and conduct multilocus sequence typing. The highest prevalence of IE was observed for trimethoprim-sulfamethoxazole (9.4%), followed by erythromycin (8.8%), linezolid (6.8%), penicillin (6.1%), clindamycin (5.5%), vancomycin (3.8%), cefazolin (3.0%), levofloxacin (1.5%), tetracycline (0.5%), and oxacillin and gentamicin (0.0%). All cefazolin IE-positive strains carried blaZ type A, and ST25 was the most common sequence type (42.9%). For trimethoprim-sulfamethoxazole, erythromycin, and clindamycin IE, ST7 was the most common sequence type (22.7%, 26.7%, and 33.3%, respectively). ST1281 and ST188 were the predominant sequence types among strains exhibiting linezolid IE and vancomycin IE (25.0% and 33.3%, respectively). Among the 234 MSSA strains, 66.7% of ST59, 60.0% of ST25, 58.3% of ST5, and 54.2% of ST7 strains exhibited IE to at least one antimicrobial agent. Cefazolin IE was associated with blaZ type A, and ST5, ST7, ST59, and ST25 were the major sequence types associated with IE across the antimicrobial classes tested.

**IMPORTANCE:**

Methicillin-susceptible *Staphylococcus aureus* (MSSA) can show an inoculum effect on multiple antimicrobial agents, which may reduce antibiotic activity under high-burden conditions. In this study, MSSA isolates from Shanghai exhibited inoculum effects on several commonly used agents, although the overall detection rates were low. Cefazolin inoculum effect was specifically associated with blaZ type A, and several major sequence types were more likely to exhibit this phenotype. These findings improve our understanding of the epidemiology of the inoculum effect in MSSA and may help guide laboratory detection and antimicrobial treatment decisions.

## INTRODUCTION

*Staphylococcus aureus*, a major gram-positive coccus, is commonly found on human skin and mucosal surfaces, with approximately 30% of healthy individuals acting as asymptomatic carriers (1, 2). This bacterium is a leading cause of both community-acquired and healthcare-associated infections (3), particularly bacteremia, pneumonia, skin and soft tissue infections, and bone and joint infections (4, 5).

Recently, the incidence of methicillin-susceptible *S. aureus* (MSSA) infections has increased, with MSSA infections occurring at nearly twice the rate of methicillin-resistant *S. aureus* (MRSA) infections (6). Treatment of MSSA infections typically involves β-lactam antibiotics (7–9). However, recent studies have identified some MSSA strains that exhibit reduced susceptibility to these antibiotics at high inocula, a phenomenon known as the inoculum effect (IE). IE can lead to therapeutic failure, particularly in deep-seated infections or infections with a high bacterial burden (10). Therefore, further investigation of the epidemiological characteristics and clinical implications of IE is warranted.

Currently, most clinical laboratories follow established guidelines for antimicrobial susceptibility testing of MSSA strains. However, traditional methods do not detect the IE. Identification of IE requires broth microdilution testing at high inoculum (HI) concentrations, which is not routinely performed in most clinical settings. As a result, IE is often overlooked, potentially leading to inappropriate treatment selection.

To date, no studies have reported the prevalence of IE in MSSA strains in China.

In this study, we evaluated the prevalence of IE to cefazolin and other antimicrobial agents among MSSA strains in Shanghai and characterized IE-positive strains via whole-genome sequencing (WGS), thereby providing evidence to inform clinical treatment decisions.

## MATERIALS AND METHODS

### Strain collection and identification

We analyzed 234 nonduplicate MSSA isolates consecutively collected from 15 hospitals across Shanghai between April and December 2023. Isolates were recovered from diverse clinical specimen types, including blood, pus, sputum, urine, and sterile body fluids, representing both invasive and non-invasive infections. Species identification was confirmed by matrix-assisted laser desorption/ionization time-of-flight mass spectrometry (MALDI-TOF MS). MSSA status was defined as an oxacillin MIC ≤2 µg/mL according to the Clinical and Laboratory Standards Institute (CLSI) M100 (34th ed.) breakpoints (11). To ensure epidemiological independence, only the first isolate from each unique patient was included; subsequent isolates from the same patient were excluded. Participating hospitals were requested to submit all MSSA isolates meeting the inclusion criteria during the study period. To maintain purity and viability, isolates were subcultured on sheep blood agar plates at 35°C for 24 h, purified, and stored at –80°C in appropriate cryopreservatives until further use.

### Minimum inhibitory concentration testing of antimicrobial agents

Minimum inhibitory concentrations (MICs) were determined by broth microdilution in Mueller–Hinton broth (MHB), supplemented as appropriate, according to CLSI M100 (34th ed.) guidelines. The MIC was defined as the lowest antimicrobial concentration that inhibited visible bacterial growth after 16–18 h of incubation at 35°C ± 2°C. Inocula were prepared from fresh 18–24 h sheep blood agar cultures. Two to three colonies were suspended in sterile diluent and adjusted to a 1.0 McFarland standard. For the standard inoculum (SI), 1 mL of the adjusted suspension was diluted in 9 mL sterile saline, and 1 mL of this dilution was further diluted in 9 mL sterile saline. Subsequently, 2 mL of the final dilution was added to 10 mL MHB to yield a final concentration of approximately 5 × 10^5 CFU/mL. For the high inoculum (HI), 2 mL of the adjusted suspension was added directly to 10 mL MHB to achieve a final concentration of approximately 5 × 10^7 CFU/mL. A 100-µL aliquot of each inoculum was dispensed into 96-well microdilution plates containing serial twofold dilutions of 11 antimicrobial agents: cefazolin (Cfz), penicillin (Pen), oxacillin (Oxa), trimethoprim-sulfamethoxazole (Sxt), erythromycin (Ery), clindamycin (Da), linezolid (Lzn), gentamicin (Gen), tetracycline (Tet), levofloxacin (Lev), and vancomycin (Van). Plates were incubated at 35°C for 18 h, and MICs were independently interpreted by two clinical microbiology technologists. *S. aureus* ATCC 29213 was included in each experiment as a quality control strain.

### Definition of inoculum effect

For cefazolin, IE was defined as an MIC ≥16 µg/mL under high-inoculum (HI; 5 × 10^7 CFU/mL) conditions and ≤8 µg/mL under standard inoculum (SI; 5 × 10^5 CFU/mL) conditions, in accordance with established definitions in the literature (12).

For all other antimicrobial agents, IE was defined as a ≥8-fold increase in MIC at HI compared with SI, consistent with widely adopted definitions in contemporary IE research (13–19).

### Whole-genome sequencing

Whole-genome sequencing (WGS) was performed on MSSA strains exhibiting the inoculum effect to identify β-lactamase types and determine multilocus sequence typing (MLST) profiles. Genomic DNA was extracted using the Qiagen DNeasy Blood & Tissue Kit. Sequencing libraries were prepared using the Nextera XT DNA Library Preparation Kit according to the manufacturer’s instructions. Paired-end sequencing (150 bp reads) was performed on a DNBSEQ PE150 platform. MLST was performed by querying assembled contigs against the *Staphylococcus aureus* scheme on the PubMLST website (https://pubmlst.org). *blaZ* genotyping was performed using the β-lactamase analysis service provided by the Center for Genomic Epidemiology (https://www.genomicepidemiology.org/services/). Classification of blaZ variants was based on amino acid positions 128 and 216, which correspond to residues 119 and 207 in the signal peptide, respectively.

### Statistical analysis

Categorical variables were analyzed using the *χ*^2^ test or Fisher’s exact test. All analyses were conducted using R (v4.2.1), and all tests were two-tailed. Statistical significance was set at *P* < 0.05. To ensure reliability and reproducibility, all statistical analyses were performed in independent laboratories.

## RESULTS

### Prevalence of IE in MSSA strains

This study evaluated IE in 234 MSSA strains collected from 15 microbiology laboratories in Shanghai and tested their susceptibility to 11 antimicrobial agents. Among the blaZ-negative strains, the highest IE rate was observed for clindamycin (6.3%), followed by penicillin (6.1%), erythromycin (3.6%), and trimethoprim-sulfamethoxazole (3.0%). No IE was detected for the remaining antimicrobial agents. Among blaZ-positive strains, the highest IE rate was recorded for trimethoprim-sulfamethoxazole (10.5%), followed by erythromycin (9.9%), linezolid (8.0%), and clindamycin (5.4%). The IE rates for vancomycin, cefazolin, levofloxacin, and tetracycline were 4.5%, 3.5%, 2.3%, and 0.6%, respectively. No IE was detected for the remaining antimicrobial agents (Table 1).

**TABLE 1:** Incidence of the inoculum effects (IE) for different antimicrobial agents and corresponding sequence types

Antimicrobial agent	Overall IE rate (%)	IE rate among *blaZ-*negative strains (%)	IE rate among *blaZ*-positive strains (%)	STs among IE-positive strains
Cefazolin (Cfz)	3.0 (7/234)	0.0 (0/33)	3.5 (7/201)	ST5 (1), ST7 (1), ST2990 (1), ST8256 (1), ST25 (3)
Oxacillin (Oxa)	0.0 (0/234)	0.0 (0/33)	0.0 (0/201)	None
Penicillin (Pen)	6.1 (2/33)	6.1 (2/33)	NA^*a*^	ST398 (1), ST59 (1)
Trimethoprim-sulfamethoxazole (Sxt)	9.4 (22/233)	3.0 (1/33)	10.5 (21/200)	ST398 (1), ST7 (5), ST5 (3), ST188 (3), ST1281 (3), ST630 (2), ST2990 (2), ST6 (1), ST72 (1), ST8256 (1)
Erythromycin (Ery)	8.8 (15/170)	3.6 (1/28)	9.9 (14/142)	ST5 (2), ST7 (4), ST22 (1), ST1 (1), ST6 (2), ST15 (1), ST1281 (2), ST573 (1), ST5459 (1)
Clindamycin (Da)	5.5 (12/218)	6.3 (2/32)	5.4 (10/186)	ST5 (3), ST59 (1), ST7 (4), ST1 (1), ST22 (1), ST2315 (1), ST8178 (1)
Linezolid (Lzn)	6.8 (16/234)	0.0 (0/33)	8.0 (16/201)	ST1281 (4), ST15 (3), ST188 (1), ST1 (1), ST6 (2), ST59 (2), ST7 (1), ST630 (1), ST965 (1)
Gentamicin (Gen)	0.0 (0/225)	0.0 (0/32)	0.0 (0/193)	None
Tetracycline (Tet)	0.5 (1/196)	0.0 (0/31)	0.6 (1/165)	ST7 (1)
Levofloxacin (Lev)	1.5 (3/205)	0.0 (0/30)	1.7 (3/175)	ST5 (1), ST9256 (1), ST398 (1)
Vancomycin (Van)	3.8 (9/234)	0.0 (0/33)	4.5 (9/201)	ST5 (1), ST6 (1), ST15 (1), ST188 (2), ST398 (1), ST3075 (1), ST8256 (1), ST630 (1)

^*a*^ NA, not assessed among blaZ-positive isolates.

Among the 73 MSSA strains exhibiting IE in this study, most (84.9%, 62/73) showed IE to only one antimicrobial agent, 11.0% (8/73) showed IE to two agents, and 4.1% (3/73) showed IE to three agents. No strains showed IE to four or more antimicrobial agents.

### Association between blaZ gene types and cefazolin MIC distribution in MSSA strains

**Fig 1:**
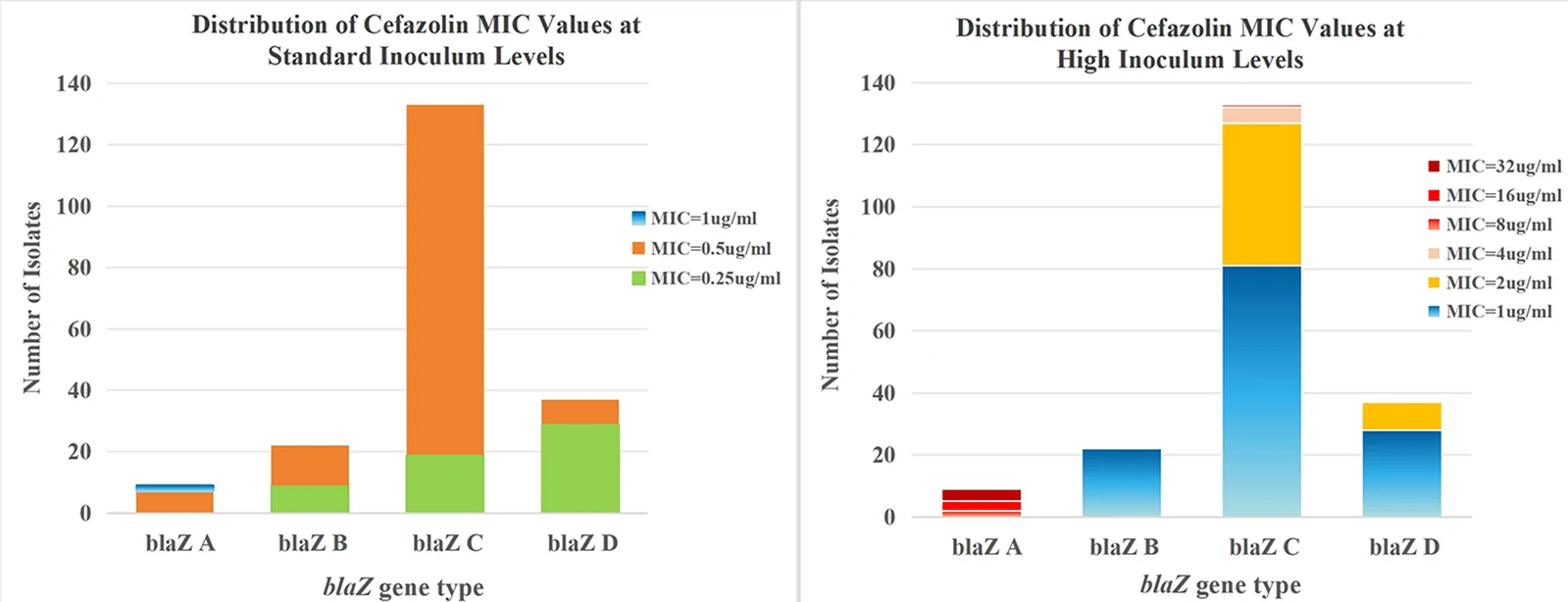
Association between blaZ type and cefazolin MIC distribution at standard and high inocula among 201 *blaZ*-positive strains. Color bars indicate cefazolin MIC values determined at standard (~10⁵ CFU/mL) and high (~10⁷ CFU/mL) inocula: green, 0.25 μg/mL; brown, 0.5 μg/mL; blue, 1 μg/mL; yellow, 2 μg/mL; pink, 4 μg/mL; light red, 8 μg/mL; red, 16 μg/mL; and dark red, 32 μg/mL.

Among the 201 blaZ-positive strains, type C was the most prevalent (66.2%, 133/201), followed by type D (18.4%, 37/201), type B (10.9%, 22/201), and type A (4.5%, 9/201). All seven cefazolin IE-positive strains belonged to type A. At SI, MICs for type A, type B, type C, and type D strains were all ≤1 µg/mL. At HI, MICs for type A strains ranged from 8 to 32 μg/mL, whereas those for type B, type C, and type D strains remained ≤4 µg/mL (Fig. 1; Table S2).

### Distribution of inoculum effect prevalence among major STs and antimicrobial agents

The 234 MSSA strains were classified into 41 STs, of which 73 IE-positive strains were distributed across 22 STs. Among the 41 STs, ST59 had the highest proportion of IE-positive strains (66.7%), followed by ST25 (60.0%), ST5 (58.3%), and ST7 (54.2%). Among the 22 IE-positive STs, ST25 was the most common ST associated with cefazolin IE (42.9%), while ST7 was the predominant ST associated with trimethoprim-sulfamethoxazole, erythromycin, and clindamycin IE, accounting for 22.7%, 26.7%, and 33.3%, respectively. ST1281 was the most common ST associated with linezolid IE (25.0%), and ST188 was the most common ST associated with vancomycin IE (22.2%) (Table 2).

**TABLE 2:** Distribution of inoculum effect prevalence among major sequence types and antimicrobial agents

ST	No. of IE-positive strains (*n* ≥ 3)	Total no. of strains	Percentage of IE-positive strains (%)	Antimicrobial agents associated with IE-positive strains, *n*
ST7	13	24	54.2	Trimethoprim-sulfamethoxazole (3), erythromycin (3), clindamycin (3), cefazolin (1), trimethoprim-sulfamethoxazole +erythromycin (1), trimethoprim-sulfamethoxazole + tetracycline (1), and clindamycin + linezolid (1)
ST5	7	12	58.3	Trimethoprim-sulfamethoxazole (2), erythromycin (1), clindamycin (1), vancomycin (1), trimethoprim-sulfamethoxazole + cefazolin (1), and clindamycin + erythromycin (1)
ST6	6	18	33.3	Erythromycin (2), linezolid (2), trimethoprim-sulfamethoxazole (1), and vancomycin (1)
ST188	6	22	27.3	Trimethoprim-sulfamethoxazole (3), vancomycin (2), and linezolid (1)
ST15	5	25	20.0	Linezolid (3), erythromycin (1), and vancomycin (1)
ST1281	5	13	38.5	Trimethoprim-sulfamethoxazole + linezolid (2), linezolid (1), erythromycin (1), and trimethoprim-sulfamethoxazole + erythromycin + linezolid (1)
ST59	4	6	66.7	Linezolid (2), clindamycin (1), and clindamycin + penicillin (1)
ST398	4	28	14.3	Trimethoprim-sulfamethoxazole (1), vancomycin (1), penicillin (1), and levofloxacin (1)
ST630	4	11	36.4	Trimethoprim-sulfamethoxazole (2), vancomycin (1), and linezolid (1)
ST1	3	7	42.9	Linezolid (1), clindamycin (1), and erythromycin (1)
ST25	3	5	60.0	Cefazolin (3)

## DISCUSSION

Penicillins and cefazolin are the preferred treatments for MSSA infections. Although cefazolin inoculum-dependent MIC elevation strains have been widely reported in *in vitro* studies, their impact on clinical treatment outcomes remains controversial. Some studies suggest that IE is associated with treatment failure and increased mortality (13, 20, 21), whereas others have failed to identify a significant correlation between IE and clinical outcomes (22, 23). These discrepancies may be related to differences in sample size, infection type, and strain characteristics (24, 25). Another often overlooked issue is the lack of a standardized definition of IE. Currently, three main definitions of β-lactam IE are used. One defines IE in MSSA as a cefazolin MIC of ≥16 µg/mL at HI and ≤8 µg/mL at SI (12). Another defines IE in MSSA as a ≥4-fold difference in MIC between HI (5 × 10⁷ CFU/mL) and SI (5 × 10⁵ CFU/mL) (26). A third definition, used for gram-negative bacilli, defines IE as a ≥8-fold MIC ratio at HI relative to SI (14). Therefore, the criteria used to define IE may influence the interpretation of clinical outcomes.

This study represents the first *in vitro* phenotypic assessment of IE across multiple antimicrobial agents in MSSA strains from Shanghai. The detection rate of cefazolin IE was 3.0% (7/234), whereas detection rates for other antimicrobial agents ranged from 0.0% to 9.4%. Reported cefazolin IE rates vary substantially by region, ranging from 0% to 27.9% in North America (27), 40.0% in Latin America (28), 20.0% in Europe (29), 20.1% in South Korea (30), and 0.0% in Japan (12). A French study reported an oxacillin IE rate of 35.6% (77/218) among MSSA isolates from patients with infective endocarditis, although only five strains (2.3%) had MIC values exceeding 2 μg/mL at HI, meeting the CLSI resistance breakpoint (21). In the present study, 1.3% (3/234) of strains showed oxacillin MIC values exceeding 2 μg/mL at HI. Additionally, this study represents the first systematic evaluation of IE in MSSA strains for nine other antimicrobial agents. Although the overall detection rate was low, IE was observed across multiple drugs.

WGS was performed to investigate the role of the blaZ gene in the IE among MSSA strains. The results showed that cefazolin IE occurred exclusively in strains carrying the blaZ type A β-lactamase. Among these, two blaZ-A MSSA strains exhibited IE to three antimicrobial agents, including cefazolin, trimethoprim-sulfamethoxazole, and either levofloxacin or vancomycin, whereas no such pattern was observed among blaZ-negative strains.

Collectively, these findings suggest a potential association between the blaZ genotype and the cefazolin-specific inoculum effect, possibly reflecting functional polymorphisms within the blaZ gene (31–34). In contrast, the mechanisms underlying IE to non-β-lactam agents remain unclear and warrant further investigation. Previous studies have identified four blaZ subtypes, A through D (27, 29, 30), and several studies have demonstrated an association between blaZ subtype and cefazolin IE (35–37). Previous research has also shown that in North America and Latin America, blaZ type C (53.7% and 37%, respectively) and type A (44.4% and 33%, respectively) are the predominant subtypes in MSSA isolates (28). A key finding of the present study was that all cefazolin IE-positive strains carried the blaZ type A subtype. Of note, 77.8% (7/9) of blaZ-type A strains exhibited cefazolin IE, with high-inoculum (HI) MICs ≥16 µg/mL and HI-to-standard inoculum (SI) MIC ratios ≥16-fold; three isolates showed increases of up to 64-fold. Although blaZ-positive strains tended to show higher IE rates to non-β-lactam agents (except clindamycin), these differences were not statistically significant (*P* > 0.05), a pattern consistent with that observed for β-lactam antibiotics. While the association between blaZ type A and the cefazolin inoculum effect has been reported previously, our findings confirm this relationship and extend it by linking cefazolin IE to specific circulating sequence types. In contrast, the observations regarding non-β-lactam agents remain descriptive, pending further mechanistic and clinical validation.

To further characterize the molecular epidemiological features of IE-positive MSSA strains in Shanghai, MLST was performed. The results showed that cefazolin IE-positive strains were most commonly associated with ST25, which accounted for 42.9% (3/7) of these strains. All three ST25 strains carried the blaZ type A gene and exhibited cefazolin IE, suggesting that ST25 may be the predominant clonal lineage associated with this phenotype in the region. In contrast, for other antimicrobial agents, the predominant ST accounted for less than 35% of IE-positive strains, and no clear association between ST and IE was observed. This finding differs from the distribution reported in North America, where ST8 (associated with blaZ type C) and ST30 (associated with blaZ type A) predominate (12), highlighting substantial interregional variation. In this study, the proportion of strains exhibiting IE to at least one antimicrobial agent was relatively high among ST5, ST7, ST59, and ST25 strains, ranging from 54.2% to 66.7%, suggesting that these STs may be more likely to exhibit IE. The mechanisms underlying this tendency warrant further investigation.

This study has several limitations. First, we did not perform direct genetic validation of the observed association between blaZ type A and the cefazolin IE. Although our findings suggest a strong phenotypic correlation, allelic exchange experiments, such as replacing blaZ type A with alternative genotypes, would be required to definitively establish causality. In addition, all data were derived from *in vitro* assays; thus, the clinical relevance of these findings remains uncertain and requires validation in animal models of infection to determine whether this phenotype contributes to treatment failure *in vivo*. Second, the isolates were collected from a geographically restricted region and may not fully represent the broader MSSA population in Shanghai. Third, the relatively small number of cefazolin IE-positive isolates (*n* = 7) limits statistical power and constrains the generalizability of our conclusions. Fourth, the mechanisms underlying dual- and triple-drug IE phenotypes remain incompletely understood and warrant further investigation. Finally, IE was assessed across multiple antimicrobial classes and summarized as a composite phenotype. Given that IE is mechanistically heterogeneous and drug-specific, this approach may obscure biologically meaningful genotype-phenotype relationships. Future research should adopt a class-specific approach to evaluating the IE, incorporating detailed mechanistic analyses rather than relying solely on aggregated multidrug IE phenotypes.

In conclusion, this study confirms the association between blaZ type A and cefazolin IE and extends this relationship to specific circulating sequence types within a regional MSSA population. In contrast, the mechanisms underlying IE to non-β-lactam agents remain incompletely understood. These findings are descriptive and highlight clonal lineages that may be predisposed to inoculum-dependent phenotypic shifts. Large-scale, targeted genetic manipulation and outcome-linked clinical studies are required to validate these observations and to determine whether IE should inform therapeutic decision-making or targeted surveillance strategies

## Data Availability

The assembled genome sequences and their corresponding annotated GenBank files have been deposited in NCBI GenBank under BioProject PRJNA1473691.

## SUPPLEMENTAL MATERIAL

Table S1 spectrum.00333-26-s0001.docx MIC values, HI/SI ratios, blaZ genotypes, and associated sequence types of inoculum effect-positive isolates under standard and high inoculum conditions.

Table S2 spectrum.00333-26-s0002.docx blaZGenotypes, cefazolin MICs at standard and high inoculum, and HI/SI ratios among 201 blaZ‑positive isolates.

ASM does not own the copyrights to Supplemental Material that may be linked to, or accessed through, an article. The authors have granted ASM a non-exclusive, world-wide license to publish the Supplemental Material files. Please contact the corresponding author directly for reuse.
