# Supplementary material for: The inoculum effect of methicillin-susceptible Staphylococcus aureus on cefazolin and other antimicrobial agents
Source: Microbiol Spectr. 2026 Jul 23;14(9):e00333-26. doi: 10.1128/spectrum.00333-26 (PMC13532247; doi:10.1128/spectrum.00333-26)
Supplement: Supplementary file 1 [file spectrum.00333-26-s0001.docx]

**Supplementary Table S1**

MIC Values, HI/SI Ratios, *blaZ* Genotypes, and Associated Sequence Types of Inoculum Effect-Positive Isolates Under Standard and High Inoculum Conditions

| Sample No. | cefazolin (MICs) | | | penicillin (MICs) | | | trimethoprim-sulfamethoxazole (MICs) | | | erythromycin (MICs) | | | clindamycin (MICs) | | | linezolid (MICs) | | | tetracycline (MICs) | | | vancomycin (MICs) | | | levofloxacin (MICs) | | | *blaZ* Gene Types | STs |
| --- | --- | --- | --- | --- | --- | --- | --- | --- | --- | --- | --- | --- | --- | --- | --- | --- | --- | --- | --- | --- | --- | --- | --- | --- | --- | --- | --- | --- | --- |
|  | SI | HI | HI/SI  ratios | SI | HI | HI/SI  ratios | SI | HI | HI/SI  ratios | SI | HI | HI/SI  ratios | SI | HI | HI/SI  ratios | SI | HI | HI/SI  ratios | SI | HI | HI/SI  ratios | SI | HI | HI/SI  ratios | SI | HI | HI/SI  ratios |  |  |
| RJ074 | 0.25 | 1 | 4 | 0.03 | 0.25 | 8 | 0.125 | 0.25 | 2 | 0.125 | 0.25 | 2 | 0.125 | 0.25 | 2 | 1 | 4 | 4 | 0.5 | 0.5 | 1 | 0.5 | 1 | 2 | 0.125 | 0.25 | 2 |  | 398 |
| DL012 | 0.5 | 2 | 4 | 0.03 | 0.25 | 8 | 0.125 | 0.5 | 4 | 0.25 | 0.5 | 2 | 0.125 | 1 | 8 | 0.5 | 1 | 2 | 16 | 32 | 2 | 1 | 2 | 2 | 0.25 | 0.5 | 2 |  | 59 |
| RJ075 | 0.25 | 1 | 4 | 0.06 | 0.125 | 2 | 0.125 | 1 | 8 | 0.125 | 0.25 | 2 | 0.125 | 0.25 | 2 | 0.5 | 2 | 4 | 0.5 | 1 | 2 | 0.5 | 2 | 4 | 0.5 | 1 | 2 |  | 398 |
| RJ066 | 0.25 | 0.5 | 2 | 0.03 | 0.06 | 2 | 0.125 | 0.5 | 4 | 0.125 | 1 | 8 | 0.125 | 1 | 8 | 0.5 | 1 | 2 | 0.5 | 0.5 | 1 | 0.5 | 1 | 2 | 0.125 | 0.25 | 2 |  | 5 |
| ZR003 | 0.5 | 32 | 64 | ＞4 | ＞4 | 1 | 0.125 | 0.25 | 2 | ＞16 | ＞16 | 1 | 0.125 | 0.5 | 4 | 1 | 4 | 4 | 0.5 | 1 | 2 | 0.25 | 1 | 4 | 0.25 | 0.5 | 2 | A | 25 |
| ZR004 | 0.5 | 32 | 64 | ＞4 | ＞4 | 1 | 0.125 | 0.5 | 4 | ＞16 | ＞16 | 1 | 0.125 | 0.5 | 4 | 1 | 4 | 4 | 0.5 | 1 | 2 | 0.25 | 1 | 4 | 0.25 | 1 | 4 | A | 25 |
| TR009 | 0.5 | 4 | 8 | ＞4 | ＞4 | 1 | 0.125 | 0.25 | 2 | ＞16 | ＞16 | 1 | 0.25 | 0.5 | 2 | 0.5 | 2 | 4 | 0.5 | 1 | 2 | 0.25 | 2 | 8 | 0.125 | 0.5 | 4 | C | 398 |
| DH003 | 0.25 | 2 | 8 | 0.5 | ＞4 | 8 | 1 | 32 | 32 | ＞16 | ＞16 | 1 | 0.125 | 0.5 | 4 | 1 | 2 | 2 | 32 | ＞32 | 1 | 0.5 | 1 | 2 | 4 | ＞16 | 4 | D | 5 |
| RJ016 | 0.5 | 4 | 8 | ＞4 | ＞4 | 1 | 0.125 | 0.5 | 4 | 0.5 | 1 | 2 | 0.125 | 0.5 | 4 | 0.5 | 8 | 16 | 0.5 | 1 | 2 | 0.5 | 1 | 2 | 0.125 | 0.25 | 2 | C | 965 |
| RJ059 | 0.25 | 2 | 8 | 4 | ＞4 | 1 | 0.5 | 32 | 64 | ＞16 | ＞16 | 1 | ＞16 | ＞16 | 1 | 0.5 | 2 | 4 | 32 | 32 | 1 | 1 | 2 | 2 | 16 | 16 | 1 | C | 188 |
| YD001 | 0.5 | 16 | 32 | ＞4 | ＞4 | 1 | 0.125 | 1 | 8 | 0.25 | 1 | 4 | 0.125 | 0.5 | 4 | 1 | 2 | 2 | 0.5 | 1 | 2 | 0.5 | 1 | 2 | 0.125 | 1 | 8 | A | 5 |
| RJ035 | 1 | 16 | 16 | ＞4 | ＞4 | 1 | 0.125 | 0.5 | 4 | ＞16 | ＞16 | 1 | 0.125 | 0.5 | 4 | 0.5 | 1 | 2 | 0.5 | 1 | 2 | 0.5 | 1 | 2 | 0.125 | 0.5 | 4 | A | 25 |
| RJ022 | 1 | 32 | 32 | 4 | ＞4 | 1 | 0.125 | 1 | 8 | 0.25 | 1 | 4 | 0.125 | 0.5 | 4 | 1 | 2 | 2 | 32 | ＞32 | 1 | 0.5 | 1 | 2 | 0.125 | 0.25 | 2 | A | 2990 |
| RJ032 | 0.5 | 16 | 32 | 4 | ＞4 | 1 | 0.5 | ＞32 | 64 | ＞16 | ＞16 | 1 | 0.125 | 0.5 | 4 | 0.5 | 2 | 4 | 0.5 | 2 | 4 | 0.5 | 4 | 8 | 0.125 | 0.25 | 2 | A | 8256 |
| RJ094 | 0.25 | 2 | 8 | 0.25 | ＞4 | 16 | 0.125 | 0.25 | 2 | 0.125 | 1 | 8 | 0.125 | 0.5 | 4 | 0.5 | 2 | 4 | 0.5 | 0.5 | 1 | 0.25 | 1 | 4 | 0.25 | 0.5 | 2 | D | 5 |
| RJ084 | 0.5 | 32 | 64 | ＞4 | ＞4 | 1 | 0.125 | 0.25 | 2 | 0.25 | 0.5 | 2 | 0.125 | 0.5 | 4 | 0.5 | 2 | 4 | 0.5 | 0.5 | 1 | 0.5 | 1 | 2 | 0.125 | 0.25 | 2 | A | 7 |
| RJ005 | 0.5 | 2 | 4 | 2 | ＞4 | 2 | 0.5 | 32 | 64 | ＞16 | ＞16 | 1 | ＞16 | ＞16 | 1 | 0.5 | 2 | 4 | 32 | 32 | 1 | 0.5 | 1 | 2 | 16 | 16 | 1 | C | 188 |
| RJ044 | 0.5 | 1 | 2 | 4 | ＞4 | 1 | 0.125 | ＞32 | 256 | 0.125 | 0.5 | 4 | 0.125 | 0.25 | 2 | 0.5 | 1 | 2 | 0.5 | 0.5 | 1 | 0.5 | 1 | 2 | 0.125 | 0.125 | 1 | C | 630 |
| FZY001 | 0.25 | 1 | 4 | 0.25 | ＞4 | 16 | 0.125 | 1 | 8 | 0.125 | 1 | 8 | 0.125 | 0.5 | 4 | 0.5 | 4 | 8 | 0.5 | 1 | 2 | 0.5 | 2 | 4 | 0.5 | 1 | 2 | D | 1281 |
| JY017 | 0.5 | 1 | 2 | 4 | ＞4 | 1 | 0.125 | 1 | 8 | 0.25 | 1 | 4 | 0.125 | 0.5 | 4 | 1 | 4 | 4 | 0.5 | 2 | 4 | 0.5 | 1 | 2 | 0.125 | 0.25 | 2 | C | 2990 |
| TR001 | 0.5 | 2 | 4 | 4 | ＞4 | 1 | 0.5 | 32 | 64 | ＞16 | ＞16 | 1 | ＞16 | ＞16 | 1 | 1 | 4 | 4 | 0.5 | 2 | 4 | 0.5 | 1 | 2 | 0.125 | 0.5 | 4 | C | 7 |
| TR013 | 0.5 | 1 | 2 | 4 | 4 | 1 | 0.125 | 1 | 8 | 0.25 | 1 | 4 | 0.125 | 0.5 | 4 | 1 | 4 | 4 | 0.5 | 1 | 2 | 0.5 | 2 | 4 | 0.125 | 0.25 | 2 | C | 6 |
| TR014 | 0.5 | 2 | 4 | 4 | ＞4 | 1 | 1 | 32 | 32 | ＞16 | ＞16 | 1 | ＞16 | ＞16 | 1 | 1 | 2 | 2 | 0.5 | 2 | 4 | 0.5 | 1 | 2 | 4 | 16 | 4 | C | 7 |
| TR016 | 0.5 | 2 | 4 | 4 | ＞4 | 1 | 2 | 32 | 16 | ＞16 | ＞16 | 1 | ＞16 | ＞16 | 1 | 1 | 4 | 4 | 0.5 | 1 | 2 | 0.5 | 1 | 2 | 0.125 | 0.5 | 4 | C | 7 |
| BZX003 | 0.5 | 1 | 2 | 2 | ＞4 | 2 | 0.125 | 2 | 16 | 0.25 | 2 | 8 | 0.125 | 0.5 | 4 | 1 | 2 | 2 | 0.5 | 1 | 2 | 0.5 | 1 | 2 | 0.125 | 0.5 | 4 | C | 7 |
| PN004 | 0.25 | 1 | 4 | 0.25 | ＞4 | 16 | 0.125 | 2 | 16 | 0.25 | 1 | 4 | 0.125 | 0.5 | 4 | 0.5 | 8 | 16 | 0.5 | 1 | 2 | 0.5 | 1 | 2 | 0.5 | 1 | 2 | D | 1281 |
| SY003 | 0.25 | 1 | 4 | 0.5 | ＞4 | 8 | 0.125 | 1 | 8 | ＞16 | ＞16 | 1 | 0.125 | 0.5 | 4 | 1 | 2 | 2 | 0.5 | 2 | 4 | 0.5 | 1 | 2 | 0.125 | 0.5 | 4 | D | 5 |
| SY004 | 0.5 | 1 | 2 | 2 | ＞4 | 2 | 0.125 | 1 | 8 | 0.25 | 0.5 | 2 | 0.125 | 0.25 | 2 | 1 | 2 | 2 | 16 | 16 | 1 | 0.25 | 1 | 4 | 0.125 | 0.25 | 2 | C | 630 |
| RJ015 | 0.5 | 1 | 2 | 2 | ＞4 | 2 | 0.125 | ＞32 | 256 | ＞16 | ＞16 | 1 | ＞16 | ＞16 | 1 | 0.5 | 2 | 4 | 32 | 32 | 1 | 0.5 | 1 | 2 | 16 | 16 | 1 | C | 188 |
| DL002 | 0.25 | 1 | 4 | 1 | ＞4 | 4 | 1 | 32 | 32 | 0.25 | 0.5 | 2 | 0.125 | 0.5 | 4 | 1 | 2 | 2 | 0.5 | 2 | 4 | 0.5 | 1 | 2 | 0.125 | 0.5 | 4 | B | 72 |
| RJ048 | 0.25 | 1 | 4 | 0.25 | ＞4 | 16 | 0.125 | 1 | 8 | 0.25 | 1 | 4 | 0.125 | 0.5 | 4 | 0.5 | 4 | 8 | 0.5 | 1 | 2 | 0.5 | 1 | 2 | 0.5 | 2 | 4 | D | 1281 |
| RJ053 | 0.5 | 2 | 4 | 4 | ＞4 | 1 | 1 | ＞32 | 32 | 0.25 | 1 | 4 | 0.125 | 0.5 | 4 | 0.5 | 1 | 2 | 0.5 | 4 | 8 | 0.5 | 1 | 2 | 0.125 | 0.25 | 2 | C | 7 |
| TR019 | 0.5 | 1 | 2 | 4 | ＞4 | 1 | 0.125 | 0.25 | 2 | 0.25 | 2 | 8 | 0.125 | 0.5 | 4 | 1 | 4 | 4 | 16 | ＞32 | 2 | 0.5 | 1 | 2 | 0.125 | 0.5 | 4 | C | 7 |
| TR020 | 0.5 | 1 | 2 | 1 | ＞4 | 4 | 0.125 | 0.25 | 2 | 0.125 | 1 | 8 | 0.125 | 0.5 | 4 | 0.5 | 2 | 4 | 0.5 | 1 | 2 | 0.5 | 1 | 2 | 0.125 | 0.25 | 2 | B | 22 |
| TR021 | 0.5 | 1 | 2 | 2 | ＞4 | 2 | 0.125 | 0.5 | 4 | 0.125 | 1 | 8 | 0.25 | 1 | 4 | 0.5 | 2 | 4 | 0.5 | 2 | 4 | 0.5 | 2 | 4 | 8 | ＞16 | 2 | C | 1 |
| TR022 | 0.5 | 1 | 2 | 4 | ＞4 | 1 | 0.125 | 0.5 | 4 | 0.125 | 1 | 8 | 0.125 | 0.5 | 4 | 0.5 | 2 | 4 | 0.5 | 2 | 4 | 0.25 | 1 | 4 | 0.25 | 0.5 | 2 | C | 7 |
| TR024 | 0.25 | 1 | 4 | 0.5 | ＞4 | 8 | 0.125 | 0.25 | 2 | 0.125 | 1 | 8 | 0.125 | 0.25 | 2 | 0.5 | 2 | 4 | 0.5 | 1 | 2 | 0.5 | 2 | 4 | 0.25 | 1 | 4 | B | 573 |
| PR004 | 0.5 | 2 | 4 | 4 | ＞4 | 1 | 0.125 | 0.5 | 4 | 0.25 | 2 | 8 | 0.125 | 0.5 | 4 | 1 | 4 | 4 | 0.5 | 1 | 2 | 0.5 | 1 | 2 | 0.25 | 0.5 | 2 | C | 5459 |
| SZX001 | 0.25 | 1 | 4 | 0.25 | ＞4 | 16 | 0.125 | 0.5 | 4 | 0.125 | 1 | 8 | 0.125 | 0.5 | 4 | 0.5 | 2 | 4 | 0.5 | 1 | 2 | 0.5 | 1 | 2 | 0.25 | 0.5 | 2 | D | 1281 |
| SY013 | 0.5 | 1 | 2 | 4 | ＞4 | 1 | 0.125 | 0.5 | 4 | 0.25 | 2 | 8 | 0.125 | 0.5 | 4 | 1 | 2 | 2 | 0.5 | 1 | 2 | 0.5 | 1 | 2 | 0.125 | 0.25 | 2 | C | 15 |
| RJ042 | 0.5 | 2 | 4 | 4 | ＞4 | 1 | 0.125 | 0.25 | 2 | 0.125 | 1 | 8 | 0.125 | 0.25 | 2 | 0.5 | 2 | 4 | 0.5 | 2 | 4 | 0.5 | 1 | 2 | 0.125 | 0.25 | 2 | C | 6 |
| RJ009 | 0.5 | 1 | 2 | 1 | ＞4 | 4 | 0.125 | 0.5 | 4 | 0.25 | 2 | 8 | 0.125 | 0.5 | 4 | 1 | 4 | 4 | 0.5 | 1 | 2 | 0.5 | 1 | 2 | 0.125 | 0.25 | 2 | C | 6 |
| RJ051 | 0.5 | 2 | 4 | 4 | ＞4 | 1 | 0.125 | 0.25 | 2 | 0.125 | 1 | 8 | 0.125 | 0.5 | 4 | 1 | 2 | 2 | 0.5 | 2 | 4 | 0.5 | 1 | 2 | 0.125 | 0.25 | 2 | C | 7 |
| ZR001 | 0.5 | 1 | 2 | 1 | ＞4 | 4 | 0.125 | 0.25 | 2 | ＞16 | ＞16 | 1 | 0.125 | 1 | 8 | 0.5 | 2 | 4 | 0.5 | 1 | 2 | 0.25 | 1 | 4 | 0.125 | 0.25 | 2 | B | 22 |
| RJ001 | 0.5 | 2 | 4 | 4 | ＞4 | 1 | 0.125 | 0.5 | 4 | 0.25 | 1 | 4 | 0.125 | 1 | 8 | 1 | 2 | 2 | 16 | ＞32 | 2 | 0.5 | 1 | 2 | 2 | 8 | 4 | C | 7 |
| TR017 | 0.25 | 1 | 4 | 0.5 | ＞4 | 8 | 0.125 | 0.25 | 2 | ＞16 | ＞16 | 1 | 0.25 | 2 | 8 | 0.5 | 2 | 4 | 0.5 | 1 | 2 | 0.5 | 1 | 2 | 0.25 | 0.5 | 2 | C | 1 |
| PR007 | 0.5 | 1 | 2 | 4 | ＞4 | 1 | 0.125 | 0.25 | 2 | ＞16 | ＞16 | 1 | 0.125 | 1 | 8 | 1 | 2 | 2 | 16 | ＞32 | 2 | 0.5 | 1 | 2 | 0.5 | 1 | 2 | C | 8178 |
| PR008 | 0.5 | 2 | 4 | ＞4 | ＞4 | 1 | 0.125 | 0.5 | 4 | ＞16 | ＞16 | 1 | 0.125 | 1 | 8 | 1 | 4 | 4 | 32 | 32 | 1 | 0.5 | 1 | 2 | 4 | 16 | 4 | C | 2315 |
| SY005 | 0.5 | 1 | 2 | 1 | ＞4 | 4 | 0.125 | 0.25 | 2 | 0.25 | 0.5 | 2 | 0.25 | 2 | 8 | 1 | 2 | 2 | 0.5 | 1 | 2 | 0.25 | 1 | 4 | 0.125 | 0.5 | 4 | C | 59 |
| SY006 | 0.5 | 1 | 2 | 1 | ＞4 | 4 | 0.125 | 0.25 | 2 | ＞16 | ＞16 | 1 | 0.25 | 2 | 8 | 1 | 4 | 4 | 0.5 | 2 | 4 | 0.25 | 1 | 4 | 0.25 | 1 | 4 | D | 5 |
| RJ034 | 0.5 | 1 | 2 | ＞4 | ＞4 | 1 | 0.125 | 0.5 | 4 | ＞16 | ＞16 | 1 | 0.125 | 1 | 8 | 0.5 | 1 | 2 | 16 | 32 | 2 | 0.5 | 1 | 2 | 0.5 | 2 | 4 | C | 7 |
| RJ026 | 0.25 | 1 | 4 | 0.25 | ＞4 | 16 | 0.125 | 0.25 | 2 | 0.25 | 1 | 4 | 0.25 | 4 | 16 | 1 | 2 | 2 | 16 | 32 | 2 | 0.5 | 1 | 2 | 16 | ＞16 | 1 | C | 7 |
| RJ037 | 0.5 | 1 | 2 | 4 | ＞4 | 1 | 0.125 | 0.5 | 4 | ＞16 | ＞16 | 1 | 0.125 | 1 | 8 | 0.5 | 4 | 8 | 0.5 | 1 | 2 | 0.5 | 1 | 2 | 0.125 | 0.25 | 2 | C | 7 |
| FZY002 | 0.5 | 1 | 2 | 4 | ＞4 | 1 | 0.125 | 0.5 | 4 | 0.25 | 1 | 4 | 0.125 | 0.25 | 2 | 0.5 | 4 | 8 | 0.5 | 1 | 2 | 0.5 | 2 | 4 | 0.25 | 1 | 4 | C | 1 |
| RJ018 | 0.5 | 1 | 2 | 2 | ＞4 | 2 | 0.125 | 0.5 | 4 | ＞16 | ＞16 | 1 | 0.125 | 0.25 | 2 | 0.5 | 4 | 8 | 0.5 | 1 | 2 | 0.5 | 1 | 2 | 4 | 16 | 4 | C | 630 |
| DH004 | 0.25 | 1 | 4 | 0.25 | ＞4 | 16 | 0.125 | 0.25 | 2 | 0.25 | 0.5 | 2 | 0.125 | 0.25 | 2 | 0.5 | 4 | 8 | 0.5 | 1 | 2 | 0.5 | 1 | 2 | 0.5 | 1 | 2 | D | 1281 |
| SZX004 | 0.5 | 1 | 2 | 2 | ＞4 | 2 | 0.125 | 0.25 | 2 | 0.25 | 0.5 | 2 | 0.125 | 0.25 | 2 | 0.5 | 4 | 8 | 16 | 32 | 2 | 0.25 | 1 | 4 | 0.125 | 0.25 | 2 | C | 15 |
| SY014 | 0.5 | 1 | 2 | 4 | ＞4 | 1 | 0.125 | 0.5 | 4 | 0.25 | 1 | 4 | 0.125 | 0.5 | 4 | 0.5 | 8 | 16 | 0.5 | 2 | 4 | 0.5 | 1 | 2 | 0.125 | 0.125 | 1 | C | 188 |
| RJ030 | 0.5 | 2 | 4 | 1 | ＞4 | 4 | 0.125 | 0.25 | 2 | ＞16 | ＞16 | 1 | ＞16 | ＞16 | 1 | 0.5 | 4 | 8 | 0.5 | 2 | 4 | 0.5 | 2 | 4 | 0.125 | 0.25 | 2 | D | 59 |
| RJ036 | 0.5 | 2 | 4 | 4 | ＞4 | 1 | 0.125 | 0.25 | 2 | 0.25 | 1 | 4 | 0.125 | 0.5 | 4 | 0.5 | 4 | 8 | 0.5 | 1 | 2 | 0.5 | 1 | 2 | 0.25 | 0.5 | 2 | C | 15 |
| RJ014 | 0.5 | 1 | 2 | 0.5 | ＞4 | 8 | 0.125 | 0.25 | 2 | 0.25 | 1 | 4 | 0.125 | 0.5 | 4 | 0.5 | 4 | 8 | 0.5 | 1 | 2 | 0.5 | 1 | 2 | 0.125 | 0.25 | 2 | D | 59 |
| RJ025 | 0.5 | 1 | 2 | 2 | ＞4 | 2 | 0.125 | 0.25 | 2 | 0.25 | 0.5 | 2 | 0.125 | 0.25 | 2 | 0.5 | 8 | 16 | 0.5 | 2 | 4 | 0.5 | 1 | 2 | 0.125 | 0.25 | 2 | C | 6 |
| RJ006 | 0.25 | 1 | 4 | 0.25 | ＞4 | 16 | 0.125 | 0.5 | 4 | 0.25 | 1 | 4 | 0.125 | 0.5 | 4 | 0.5 | 8 | 16 | 0.5 | 2 | 4 | 0.5 | 1 | 2 | 0.125 | 0.25 | 2 | C | 6 |
| RJ023 | 0.5 | 1 | 2 | 0.5 | ＞4 | 8 | 0.125 | 0.5 | 4 | 0.25 | 1 | 4 | 0.125 | 0.5 | 4 | 0.5 | 4 | 8 | 0.5 | 1 | 2 | 0.5 | 1 | 2 | 0.125 | 0.5 | 4 | B | 15 |
| FZY003 | 0.5 | 2 | 4 | ＞4 | ＞4 | 1 | 0.125 | 0.25 | 2 | 0.25 | 0.5 | 2 | 0.125 | 0.25 | 2 | 1 | 4 | 4 | 0.5 | 1 | 2 | 0.25 | 4 | 16 | 0.125 | 0.25 | 2 | C | 188 |
| ZR002 | 0.5 | 1 | 2 | 2 | ＞4 | 2 | 0.125 | 0.5 | 4 | 0.25 | 0.5 | 2 | 0.125 | 0.25 | 2 | 1 | 4 | 4 | 0.5 | 1 | 2 | 0.25 | 2 | 8 | 0.125 | 0.5 | 4 | C | 188 |
| TR011 | 0.5 | 2 | 4 | 4 | ＞4 | 1 | 0.125 | 0.5 | 4 | 0.25 | 1 | 4 | 0.125 | 0.25 | 2 | 1 | 2 | 2 | 0.5 | 1 | 2 | 0.5 | 4 | 8 | 0.125 | 0.25 | 2 | C | 6 |
| PR009 | 0.5 | 2 | 4 | 4 | ＞4 | 1 | 0.125 | 0.25 | 2 | 0.25 | 0.5 | 2 | 0.125 | 0.5 | 4 | 1 | 2 | 2 | 16 | 32 | 2 | 0.25 | 2 | 8 | 0.125 | 0.5 | 4 | C | 3075 |
| RJ047 | 0.5 | 1 | 2 | 2 | ＞4 | 2 | 0.125 | 0.25 | 2 | ＞16 | ＞16 | 1 | 0.125 | 0.5 | 4 | 0.5 | 1 | 2 | 0.5 | 0.5 | 1 | 0.5 | 4 | 8 | 0.125 | 0.25 | 2 | C | 630 |
| RJ071 | 0.5 | 2 | 4 | 2 | ＞4 | 2 | 0.125 | 0.25 | 2 | ＞16 | ＞16 | 1 | 0.125 | 0.25 | 2 | 0.5 | 2 | 4 | 0.5 | 1 | 2 | 0.25 | 2 | 8 | 0.125 | 0.25 | 2 | C | 15 |
| RJ077 | 0.5 | 2 | 4 | 0.5 | ＞4 | 8 | 0.125 | 0.25 | 2 | 0.25 | 0.5 | 2 | 0.125 | 0.25 | 2 | 1 | 2 | 2 | 0.5 | 1 | 2 | 0.5 | 4 | 8 | 8 | ＞16 | 2 | D | 5 |
| PN005 | 0.5 | 2 | 4 | 4 | ＞4 | 1 | 0.125 | 0.25 | 2 | 0.25 | 0.5 | 2 | 0.125 | 0.25 | 2 | 1 | 1 | 1 | 0.5 | 0.5 | 1 | 0.5 | 2 | 4 | 0.125 | 1 | 8 | C | 398 |
| RJ011 | 0.5 | 1 | 2 | 0.5 | ＞4 | 8 | 0.125 | 0.25 | 2 | 0.25 | 1 | 4 | 0.125 | 0.5 | 4 | 1 | 2 | 2 | 0.5 | 1 | 2 | 0.5 | 1 | 2 | 0.125 | 1 | 8 | B | 9156 |

SI: Standard inoculation

HI: High inoculation

STs: Sequence Types
