# Supplementary material for: The inoculum effect of methicillin-susceptible Staphylococcus aureus on cefazolin and other antimicrobial agents
Source: Microbiol Spectr. 2026 Jul 23;14(9):e00333-26. doi: 10.1128/spectrum.00333-26 (PMC13532247; doi:10.1128/spectrum.00333-26)
Supplement: Supplementary file 2 [file spectrum.00333-26-s0002.docx]

**Supplementary Table S2**

*blaZ*Genotypes, Cefazolin MICs at Standard and High Inoculum, and HI/SI Ratios Among 201 *blaZ*‑Positive Isolates

| *blaZ* gene types | Sample No. | MICs at standard inoculum | MICs at high inoculum | Ratio of MICs at High Inoculum to Standard Inoculum |
| --- | --- | --- | --- | --- |
| A | ZR003 | 0.5 | 32 | 64 |
| A | ZR004 | 0.5 | 32 | 64 |
| A | JY004 | 0.5 | 8 | 16 |
| A | PR001 | 0.5 | 8 | 16 |
| A | YD001 | 0.5 | 16 | 32 |
| A | RJ035 | 1 | 16 | 16 |
| A | RJ022 | 1 | 32 | 32 |
| A | RJ032 | 0.5 | 16 | 32 |
| A | RJ084 | 0.5 | 32 | 64 |
| B | FZY004 | 0.25 | 1 | 4 |
| B | BY001 | 0.25 | 0.5 | 4 |
| B | ZR001 | 0.5 | 0.5 | 2 |
| B | JY002 | 0.5 | 0.5 | 2 |
| B | JY012 | 0.5 | 0.5 | 2 |
| B | TR020 | 0.5 | 0.5 | 2 |
| B | TR024 | 0.25 | 0.5 | 4 |
| B | BZX004 | 0.5 | 0.5 | 2 |
| B | PN001 | 0.25 | 1 | 4 |
| B | PN003 | 0.5 | 1 | 2 |
| B | DH002 | 0.25 | 1 | 4 |
| B | DH007 | 0.5 | 1 | 2 |
| B | SY008 | 0.5 | 0.5 | 2 |
| B | RJ011 | 0.5 | 1 | 2 |
| B | RJ027 | 0.25 | 0.5 | 4 |
| B | RJ010 | 0.5 | 1 | 2 |
| B | RJ033 | 0.25 | 0.5 | 4 |
| B | DL002 | 0.25 | 0.5 | 4 |
| B | DL006 | 0.25 | 0.5 | 4 |
| B | RJ054 | 0.5 | 0.5 | 2 |
| B | RJ052 | 0.5 | 0.5 | 2 |
| B | RJ023 | 0.5 | 0.5 | 2 |
| C | RJ005 | 0.5 | 2 | 4 |
| C | RJ046 | 0.5 | 2 | 4 |
| C | RJ017 | 0.5 | 1 | 2 |
| C | RJ044 | 0.5 | 1 | 2 |
| C | RJ056 | 0.5 | 2 | 4 |
| C | DL011 | 0.5 | 2 | 4 |
| C | FZY002 | 0.5 | 1 | 2 |
| C | FZY003 | 0.5 | 2 | 4 |
| C | ZR002 | 0.5 | 0.5 | 2 |
| C | JY001 | 0.5 | 1 | 2 |
| C | JY003 | 0.5 | 2 | 4 |
| C | JY005 | 0.5 | 1 | 2 |
| C | JY007 | 0.5 | 0.5 | 2 |
| C | JY008 | 0.5 | 0.5 | 2 |
| C | RJ001 | 0.5 | 2 | 4 |
| C | JY009 | 0.5 | 1 | 2 |
| C | JY010 | 0.5 | 0.5 | 2 |
| C | JY013 | 0.5 | 0.5 | 2 |
| C | JY014 | 0.5 | 1 | 2 |
| C | JY016 | 0.5 | 0.5 | 2 |
| C | JY015 | 0.5 | 1 | 2 |
| C | JY017 | 0.5 | 0.5 | 2 |
| C | JY018 | 0.5 | 0.5 | 2 |
| C | JY019 | 0.5 | 1 | 2 |
| C | JY022 | 0.5 | 1 | 2 |
| C | JY021 | 0.5 | 1 | 2 |
| C | TR001 | 0.5 | 2 | 4 |
| C | TR004 | 0.5 | 2 | 4 |
| C | TR006 | 0.25 | 0.5 | 4 |
| C | TR007 | 0.5 | 1 | 2 |
| C | TR008 | 0.25 | 1 | 4 |
| C | TR009 | 0.5 | 4 | 8 |
| C | TR010 | 0.5 | 2 | 4 |
| C | TR011 | 0.5 | 2 | 4 |
| C | TR012 | 0.5 | 1 | 2 |
| C | TR013 | 0.5 | 1 | 2 |
| C | TR014 | 0.5 | 2 | 4 |
| C | TR015 | 0.5 | 1 | 2 |
| C | TR016 | 0.5 | 2 | 4 |
| C | TR017 | 0.25 | 1 | 4 |
| C | TR019 | 0.5 | 1 | 2 |
| C | TR021 | 0.5 | 0.5 | 2 |
| C | TR022 | 0.5 | 1 | 2 |
| C | TR023 | 0.5 | 1 | 2 |
| C | BZX001 | 0.5 | 1 | 2 |
| C | BZX002 | 0.5 | 2 | 4 |
| C | BZX007 | 0.5 | 1 | 2 |
| C | RJ018 | 0.5 | 1 | 2 |
| C | BZX008 | 0.5 | 2 | 4 |
| C | BZX003 | 0.5 | 0.5 | 2 |
| C | BZX005 | 0.5 | 1 | 2 |
| C | BZX006 | 0.5 | 0.5 | 2 |
| C | PN002 | 0.5 | 2 | 4 |
| C | RJ057 | 0.25 | 2 | 8 |
| C | PN005 | 0.5 | 2 | 4 |
| C | PN006 | 0.25 | 1 | 4 |
| C | DH001 | 0.25 | 0.5 | 4 |
| C | RJ016 | 0.5 | 4 | 8 |
| C | DH005 | 0.5 | 2 | 4 |
| C | DH008 | 0.5 | 2 | 4 |
| C | TL001 | 0.5 | 2 | 4 |
| C | PR002 | 0.5 | 1 | 2 |
| C | PR004 | 0.5 | 2 | 4 |
| C | PR005 | 0.5 | 2 | 4 |
| C | PR007 | 0.5 | 1 | 2 |
| C | RJ059 | 0.25 | 2 | 8 |
| C | PR008 | 0.5 | 2 | 4 |
| C | PR009 | 0.5 | 2 | 4 |
| C | PR010 | 0.5 | 2 | 4 |
| C | SZX007 | 0.5 | 0.5 | 2 |
| C | SZX006 | 0.25 | 0.5 | 4 |
| C | SZX005 | 0.5 | 2 | 4 |
| C | SZX004 | 0.5 | 0.5 | 2 |
| C | SZX002 | 0.5 | 1 | 2 |
| C | DL013 | 0.5 | 0.5 | 2 |
| C | YD002 | 0.25 | 0.5 | 4 |
| C | YD003 | 0.25 | 0.5 | 4 |
| C | SY001 | 0.5 | 1 | 2 |
| C | SY002 | 0.25 | 0.5 | 4 |
| C | SY004 | 0.5 | 0.5 | 2 |
| C | SY005 | 0.5 | 0.5 | 2 |
| C | SY007 | 0.5 | 1 | 2 |
| C | SY011 | 0.25 | 0.5 | 4 |
| C | SY012 | 0.5 | 2 | 4 |
| C | SY013 | 0.5 | 1 | 2 |
| C | SY014 | 0.5 | 1 | 2 |
| C | SY015 | 0.5 | 1 | 2 |
| C | RJ015 | 0.5 | 1 | 2 |
| C | RJ013 | 0.5 | 1 | 2 |
| C | RJ049 | 0.5 | 1 | 2 |
| C | RJ038 | 0.25 | 1 | 4 |
| C | RJ034 | 0.5 | 1 | 2 |
| C | RJ007 | 0.5 | 1 | 2 |
| C | RJ008 | 0.5 | 1 | 2 |
| C | RJ026 | 0.25 | 0.5 | 4 |
| C | RJ028 | 0.5 | 1 | 2 |
| C | RJ036 | 0.5 | 2 | 4 |
| C | RJ041 | 0.25 | 2 | 8 |
| C | RJ042 | 0.5 | 2 | 4 |
| C | RJ025 | 0.5 | 1 | 2 |
| C | RJ006 | 0.25 | 1 | 4 |
| C | RJ009 | 0.5 | 0.5 | 2 |
| C | RJ012 | 0.25 | 2 | 8 |
| C | RJ058 | 0.5 | 0.5 | 2 |
| C | DL001 | 0.5 | 1 | 2 |
| C | DL003 | 0.5 | 1 | 2 |
| C | DL009 | 0.25 | 1 | 4 |
| C | DL004 | 0.5 | 1 | 2 |
| C | DL005 | 0.5 | 2 | 4 |
| C | DL010 | 0.25 | 0.5 | 4 |
| C | DL007 | 0.5 | 1 | 2 |
| C | DL008 | 0.5 | 1 | 2 |
| C | RJ055 | 0.5 | 2 | 4 |
| C | RJ020 | 0.5 | 1 | 2 |
| C | RJ051 | 0.5 | 2 | 4 |
| C | RJ053 | 0.5 | 2 | 4 |
| C | RJ021 | 0.5 | 4 | 8 |
| C | RJ003 | 0.5 | 1 | 2 |
| C | RJ024 | 0.5 | 8 | 16 |
| C | RJ031 | 0.5 | 2 | 4 |
| C | RJ037 | 0.5 | 1 | 2 |
| C | RJ047 | 0.5 | 1 | 2 |
| C | RJ045 | 0.5 | 1 | 2 |
| C | RJ088 | 0.5 | 2 | 4 |
| C | RJ096 | 0.5 | 4 | 8 |
| C | RJ095 | 0.5 | 4 | 8 |
| C | RJ093 | 0.5 | 2 | 4 |
| C | RJ092 | 0.5 | 2 | 4 |
| C | RJ073 | 0.5 | 2 | 4 |
| C | RJ071 | 0.5 | 2 | 4 |
| C | RJ082 | 0.5 | 2 | 4 |
| C | RJ080 | 0.5 | 2 | 4 |
| C | RJ070 | 0.5 | 2 | 4 |
| D | RJ004 | 0.25 | 1 | 4 |
| D | FZY001 | 0.25 | 0.5 | 4 |
| D | BY002 | 0.5 | 0.5 | 2 |
| D | JY006 | 0.25 | 1 | 4 |
| D | JY011 | 0.25 | 0.5 | 4 |
| D | JY020 | 0.5 | 0.5 | 2 |
| D | TR002 | 0.25 | 1 | 4 |
| D | TR003 | 0.25 | 1 | 4 |
| D | TR005 | 0.25 | 1 | 4 |
| D | TR018 | 0.25 | 1 | 4 |
| D | PN004 | 0.25 | 1 | 4 |
| D | DH003 | 0.25 | 2 | 8 |
| D | DH004 | 0.25 | 1 | 4 |
| D | DH006 | 0.25 | 1 | 4 |
| D | PR003 | 0.25 | 1 | 4 |
| D | PR006 | 0.25 | 2 | 8 |
| D | SZX001 | 0.25 | 0.5 | 4 |
| D | SZX003 | 0.25 | 0.5 | 4 |
| D | SY003 | 0.25 | 0.5 | 4 |
| D | RJ043 | 0.25 | 1 | 4 |
| D | SY006 | 0.5 | 1 | 2 |
| D | SY009 | 0.5 | 2 | 4 |
| D | SY010 | 0.25 | 0.5 | 4 |
| D | RJ019 | 0.25 | 1 | 4 |
| D | RJ030 | 0.5 | 2 | 4 |
| D | RJ029 | 0.25 | 1 | 4 |
| D | RJ014 | 0.5 | 1 | 2 |
| D | RJ040 | 0.25 | 1 | 4 |
| D | RJ002 | 0.25 | 0.5 | 4 |
| D | RJ048 | 0.25 | 0.5 | 4 |
| D | RJ050 | 0.25 | 0.5 | 4 |
| D | RJ039 | 0.25 | 1 | 4 |
| D | RJ090 | 0.25 | 2 | 8 |
| D | RJ089 | 0.25 | 2 | 8 |
| D | RJ094 | 0.25 | 2 | 8 |
| D | RJ091 | 0.5 | 2 | 4 |
| D | RJ077 | 0.5 | 2 | 4 |
